# Up-regulation of SLC27A2 suppresses the proliferation and invasion of renal cancer by down-regulating CDK3-mediated EMT

**DOI:** 10.1038/s41420-022-01145-8

**Published:** 2022-08-04

**Authors:** Ning Xu, Wen Xiao, Xiangui Meng, Weiquan Li, Xuegang Wang, Xiaoping Zhang, Hongmei Yang

**Affiliations:** 1 Department of Pathogenic Biology, School of Basic Medicine, Tongji Medical College, Huazhong University of Science and Technology, Wuhan, 430030 Hubei Province China; 2 Department of Urology, Union Hospital, Tongji Medical College, Huazhong University of Science and Technology, Wuhan, 430022 China; 3 Department of Urology, The First Affiliated Hospital, School of Medicine, Xiamen University, Xiamen, Fujian China

**Keywords:** Renal cell carcinoma, Gene regulation

## Abstract

Clear cell renal cell carcinoma (ccRCC) is one of the most common malignant tumors of the urinary system. Distant metastasis is the leading cause of poor prognosis in ccRCC. However, ccRCC is found poorly responsitive to radiotherapy and chemotherapy. Effective therapeutic strategies for its metastasis remain scarce. We analyzed clinical samples and public database, for differential expression of SLC27A2 and further explored its relationship with clinical prognosis. Biochemistry and functional experiments were carried out to study the potential mechanisms of SLC27A2, CDK3, and EMT. SLC27A2 was significantly downregulated in clinical specimens and renal cancer cell lines and predicted poor prognosis. We found that specific upregulation of SLC27A2 could significantly inhibited the proliferation, migration, and invasion of renal cancer cell lines. SLC27A2 could also influence the Epithelial-mesenchymal transition (EMT) signaling pathway, linked to the progression and metastasis of renal cancer. Using whole transcriptome sequencing of SLC27A2, CDK3 was identified as a regulatory SLC27A2 target. In terms of mechanism, SLC27A2 may further inhibit the epithelial-to-mesenchymal transition by negatively regulating CDK3. Our work suggests that functional inhibition of SLC27A2-CDK3-EMT axis may be an attractive therapeutic target for metastasis of ccRCC.

## Introduction

Renal cancer is a highly prevalent malignant solid tumor of the urinary system. According to the Chinese Medical Journal of renal malignancies, approximately 77,410 and 71,676 new kidney cancer cases will be diagnosed in China and the United States, respectively, in 2022, and approximately 46,345 and 15,259 people will die from the disease [1]. CcRCC takes accounts for most of the malignant subtypes of kidney cancer [2].

Distant metastasis occurs in 30% of ccRCC patients during initial diagnosis, resulting in a poor prognosis [3]. The molecular mechanism of renal cell carcinoma metastasis remains unclear. Therefore, it is critical to investigate the mechanism of renal cell carcinoma metastasis and identify new targets for clinical treatment.

SLC27A2, a member of the solute carrier family 27, is a critical fatty acid transporter involved in various developmental processes, including lipid biosynthesis and fatty acid breakdown [4]. SLC27A2, as a tumor suppressor gene, is associated with multiple human malignancies, for instance, lung cancer [5], ovarian cancer [6], and prostate cancer [7]. However, there are few studies on the expression of SLC27A2 and its biological behavior in renal cell carcinoma [8].

Epithelial to mesenchymal transition (EMT) is crucial in differentiating various tissues and organs [9]. EMT contributes to tissue repair, but it can also promote cancer progression through various mechanisms [10]. EMT gives cells the ability to migrate and invade, induces stem cell qualities, inhibits apoptosis and senescence, and contributes to immunosuppression [11]. Moreover, the ability of cells to move to distant organs and maintain stem cells is linked to the mesenchymal state. Therefore, exploring the EMT-related mechanisms in tumors and targeting EMT in the treatment of tumors have received much attention. However, the relationship between SLC27A2 and EMT in renal cancer has not been explored.

Cyclin-dependent kinase 3 (CDK3) encodes a cyclin-associated protein kinase family member. The protein induces S phase in cells by activating E2F transcription factor family members [12]. The protein is also associated with cyclin C and phosphorylates the retinoblastoma 1 protein to promote exit from the G_0_ stage [13]. CDK3 has been linked to EMT and the growth of cancerous tumors [14]. Whether and what role CDK3 plays in SLC27A2 affecting EMT remains unclear.

In this article, we verified the low expression level of SLC27A2 in both cell lines and renal cancer tissues. Upregulation of SLC27A2 significantly inhibited the migration and invasion abilities of renal cancer cells 786-O and CAKI. Increased SLC27A2 expression partially reversed the EMT process in ccRCC cells and downregulated the expression of CDK3. As a result, we speculated that SLC27A2 might take part in the migration and invasion of renal cancer cell by coordinating CDK3-mediated EMT in ccRCC. Mechanistic and functional studies revealed that SLC27A2 orchestrates EMT through CDK3, partially reversing this process and inhibiting tumor progression. It provided a theoretical basis for considering SLC27A2 as a potential therapeutic target for ccRCC.

## Results

### SLC27A2 was downregulated in RCC and could act as a potential biomarker for RCC

**Fig. 1:**
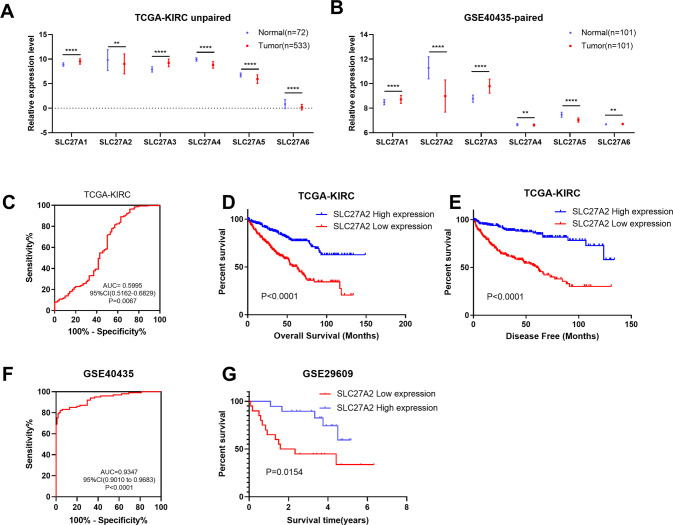
SLC27A2 was downregulated in ccRCC and could act as a potential biomarker for ccRCC. **A** Expression profiles of SLC27As mRNA in ccRCC tissues (*n* = 533) and neighboring normal tissues *n* = 72) were downloaded from TCGA-PRAD database. **B** Expression profiles of SLC27As mRNA in ccRCC tissues (*n* = 101) and neighboring normal tissues (*n* = 101) were downloaded from GSE40435-paired. **C** ROC curve of SLC27A2. **D**, **E** Kaplan–Meier curves of SLC27A2 expression. **F** ROC curve of SLC27A2 in GSE40435. **G** Kaplan–Meier curves of SLC27A2 expression.

To determine the roles that genes in the solute carrier family 27 play in the ccRCC, the relative mRNA levels of SLC27A1 to SLC27A6 were analyzed using the TCGA-KIRC database and confirmed using data from GSE40435 [15] and GSE29609 [16]. The results showed that SLC27A1 and SLC27A3 were higher-expressed in tumor samples of both sets, while SLC27A2, SLC27A4, and SLC27A5 expressions were much lower (Fig. 1A, B). Notably, SLC27A6 expressed lower in TCGA tumor samples than in GSE40435 tumor tissues. Furthermore, overall survival (OS) and disease free survival (DFS) analysis of all six candidates were analyzed using TCGA-KIRC related information. The results revealed that none of SLC27A1, 3, 4, 5, 6 possessed prognostic values for both the OS and DFS of ccRCC simultaneously (Fig. S1). Although the calculated p-value of SLC27A6 was less than 0.05, it expressed lower in tumor tissues, and the lower expression of SLC27A6 suggested better survival performance. Meaningfully, the low expression of SLC27A2 exhibited poor OS and DFS, suggesting it as an effective prognostic biomarker for ccRCC (Fig. 1D, E). In addition, data from GSE29609 were used to prove the prognostic value of SLC27A2 (Fig. 1G). ROC curves suggested that SLC27A2 could act as a diagnostic biomarker for ccRCC using TCGA-KIRC and GSE40435 data (Fig. 1C, *P* = 0.0067; Fig. 1F, *P* < 0.0001).

### Aberrant expression of SLC27A2 was significantly associated with different clinical features

**Fig. 2:**
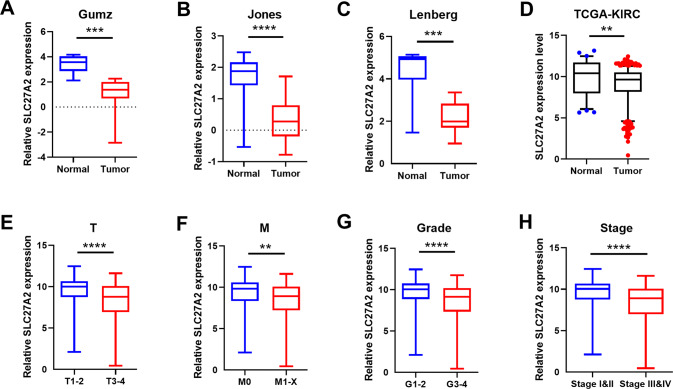
Aberrant expression of SLC27A2 was significantly associated with different clinical features. **A**–**C** SLC27A2 mRNA expression in three Oncomine subsets, including Gumz, Jones and Lenberg renal statistics, **p < 0.01, ***p < 0.001, and ****p < 0.0001. **D**–**H** SLC27A2 expression in TCGA-KIRC datasets, including cancer versus para-cancer, T stage, M stage, G stage, and TNM stage.

Using public databases, we initially confirmed that SLC27A2 expression level was lower in ccRCC than in normal kidney samples. The results showed that SLC27A2 was notably downregulated in Gumz, Jones, Lenberg, and The Cancer Genome Atlas Kidney Renal Clear Cell Carcinoma (TCGA-KIRC) data (Fig. 2A–D). In addition, SLC27A2 expression was found to be substantially linked with a variety of clinical characteristics. The higher the T stage was, the lower the SLC27A2 expressed (Fig. 2E). The expression of SLC27A2 was lower in samples with the M1 stage than those with the M0 stage (Fig. 2F). Patients in G1 or G2 grade showed higher SLC27A2 expression levels than the TNM III or IV (Fig. 2G, H). SLC27A2 was an independent predictor of ccRCC in univariate and multivariate cox proportional hazards regression analyses. Thus, SLC27A2 could be used as a diagnostic and prognostic biomarker in ccRCC (Tables 1 and 2).

**Table 1:** Univariate and multivariate analyses of risk score and patient overall survival.

Variables	Univariate analysis			Multivariate analysis^c^		
	HR^a^	95% CI^b^	*P* value	HR^a^	95% CI^b^	*P* value
Age (years) <60 vs ≥60	1.766	1.297–2.404	<0.001	1.680	1.232–2.293	0.001
Gender Female vs Male	0.965	0.707–1.318	0.825			
T stage T1 or T2 vs T3 or T4	3.043	2.245–4.124	<0.001	1.528	1.062–2.197	0.022
N stage N0 or Nx vs N1	3.554	1.871–6.748	<0.001	1.864	0.968–3.591	0.063
M stage M0 or Mx vs M1	4.369	3.197–5.971	<0.001	2.505	1.739–3.609	<0.001
Grade G1 or G2 or Gx vs G3 or G4	2.605	1.853–3.661	<0.001	1.559	1.078–2.256	0.018
SLC27A2 High vs Low	0.350	0.252–0.486	<0.001	0.520	0.367–0.738	<0.001

^a^Hazard ratio, estimated from Cox proportional hazard regression model.

^b^Confidence interval of the estimated HR.

^c^Multivariate models were adjusted for T, N, M classification, age, and gender.

**Table 2:** Univariate and multivariate analyses of risk score and patient disease free survival.

Variables	Univariate analysis			Multivariate analysis^c^		
	HR^a^	95% CI^b^	*P* value	HR^a^	95% CI^b^	*P* value
Age(years) <60 vs ≥60	1.384	0.968–1.977	0.075	1.406	0.962–2.056	0.078
Gender Female vs Male	1.469	0.985–2.193	0.060	1.026	0.677–1.554	0.905
T stage T1 or T2 vs T3 or T4	4.460	3.090–6.437	<0.001	1.872	1.224–2.864	0.004
N stage N0 or Nx vs N1	6.110	3.067–12.173	<0.001	3.043	1.458–6.348	0.003
M stage M0 or Mx vs M1	8.487	5.834–12.348	<0.001	4.568	3.004–6.947	<0.001
TNM stage I + II vs III + IV	6.579	4.408–9.820	<0.001			
Grade G1 or G2 or Gx vs G3 or G4	3.400	2.250–5.136	<0.001	2.075	1.340–3.216	0.001
SLC27A2 High vs Low	0.243	0.160–0.370	<0.001	0.378	0.242–0.589	<0.001

^a^Hazard ratio, estimated from Cox proportional hazard regression model.

^b^Confidence interval of the estimated HR.

^c^Multivariate models were adjusted for T, N, M classification, age, and gender.

### SLC27A2 was importantly downregulated in ccRCC tissues and cell lines

**Fig. 3:**
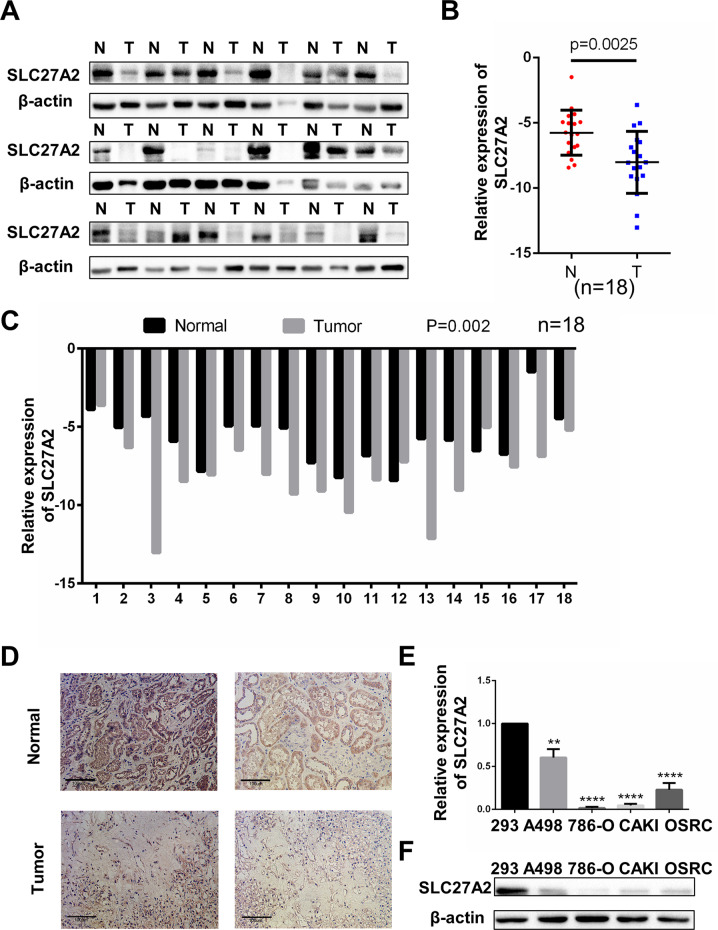
SLC27A2 was significantly downregulated in ccRCC tissues and cell lines. **A** The protein levels of SLC27A2 in ccRCC tissues and neighboring normal tissues. **B**, **C** qRT-PCR analysis of SLC27A2 mRNA expression in 18 pairs of ccRCC tissues and neighboring normal tissues. **D** The immunohistochemistry (IHC) staining for SLC27A2 in ccRCC tissues and neighboring normal tissues (Magnification: 200×). **E**, **F** The mRNA levels and protein levels in five ccRCC cell lines (A498, 786-O, CAKI, and OSRC) and normal cell line (293), *t*-test, **p < 0.01,****p < 0.0001.

Paired tissues were obtained from Wuhan Union Hospital to confirm the level of SLC27A2 expression in ccRCC tissues. The SLC27A2 expression level was lower in ccRCC tissues compared to neighboring normal tissue samples in Western blotting and qRT-PCR detection (Fig. 3A–C). The results of immunohistochemistry were similar (Fig. 3D). We also used qRT-PCR and Western blotting to show that SLC27A2 in RCC cells (A498, 786-O, Caki, and OSRC) was lower than in a normal kidney cell line (293) in both mRNA and protein levels (Fig. 3E, F). Collectively, these results confirm the fact that SLC27A2 is underexpressed in ccRCC.

### SLC27A2 functioned as an anti-oncogene and inhibited metastasis and EMT in ccRCC

**Fig. 4:**
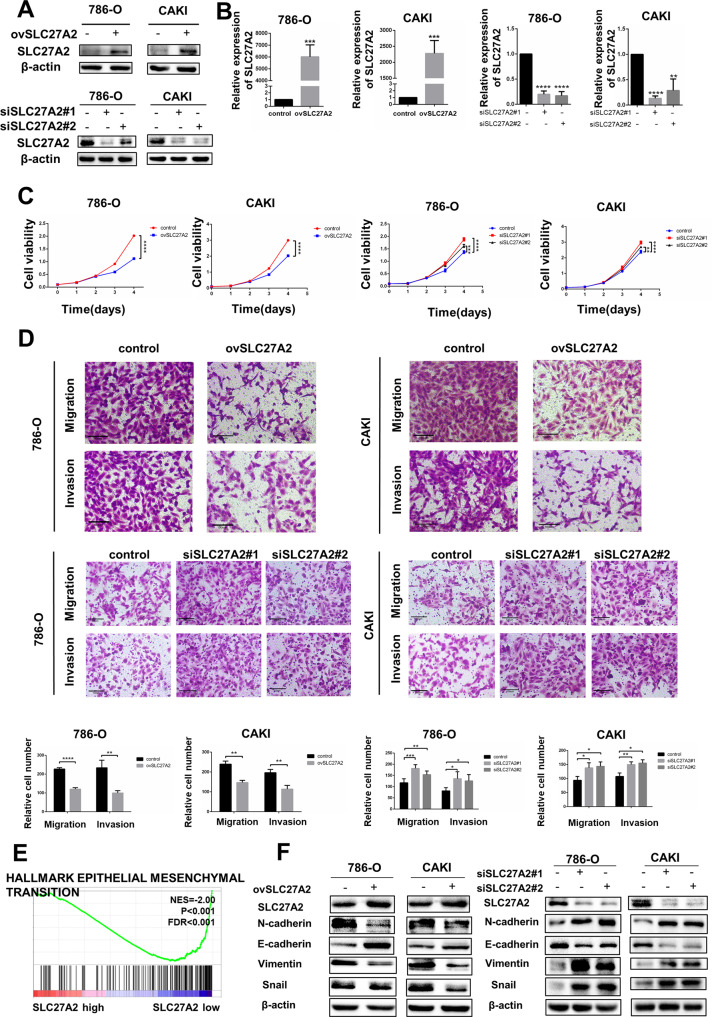
SLC27A2 functioned as an anti-oncogene and inhibited metastasis and EMT in ccRCC. **A**, **B** SLC27A2 protein and mRNA expression after overexpression or knockdown. **C** Cell growth curves of CCK8 assay for indicated cells. **D** Migration and invasion assays for indicated cells (Magnification: 200×). **E** GSEA assays for the correlation of EMT signaling pathway and mRNA level of SLC27A2 according to TCGA database. **F** The expression change of EMT markers in ccRCC cells with overexpressing or knockdown SLC27A2. *p < 0.5, **p < 0.01, ***p < 0.001, and ****p < 0.0001.

To figure out the influence of SLC27A2 to the metastasis of RCC, we performed numbers of functional tests utilizing mentioned above cell lines. SLC27A2-overexpressing lentivirus and SLC27A2 small interfering RNA (siRNA) were transfected into relevant cell lines to generate renal cancer cells with high SLC27A2 expression and low SLC27A2 expression (786-O and Caki) (Fig. 4A, B). The capacity of ccRCC cells to proliferate was assessed using the CCK8 assay. According to the findings, the proliferation rate of 786-O and Caki cells was dramatically reduced in cells overexpressing SLC27A2 (Fig. 4C). Meanwhile, the proliferation rate of 786-O and Caki cells was improved in cells when knocking down SLC27A2 (Fig. 4C).

To determine the role of SLC27A2 in cell migration, trasnwell migration experiments were performed in 786-O and Caki cells by comparing groups before and after lentiviral transfection. As expected, the migratory capability was decreased in 786-ovSLC27A2 and caki-ovSLC27A2 cells while the migratory capability was increased in 786-siSLC27A2 and caki-siSLC27A2 cells (Fig. 4D). Transwell invasion assays were also used to measure invasion capabilities. The ability of SLC27A2 overexpressing cells to invade was dramatically diminished while the ability of cancer cells with SLC27A2 koncked down to invade was dramatically rised (Fig. 4D).

**Fig. 5:**
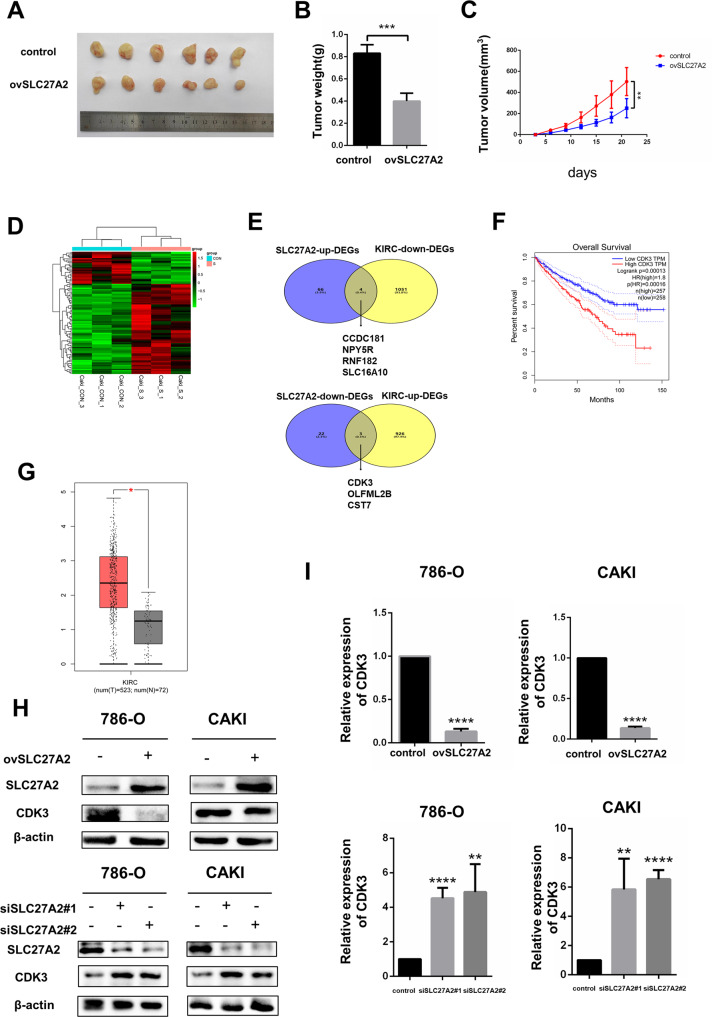
SLC27A2 could negatively regulate the expression of CDK3 in RCC. **A** Nude mice were inoculated with CAKI cells stably overexpressing SLC27A2. Images of tumors dissected from the mice at the end of experiment. **B** Tumors were weighted after excision. The data are presented as the means ± SEM. **C** The tumor size was measured every 3 days. **D** Cluster analysis heatmap based on sequencing results. **E** The upper part is the interaction of SLC27A2-up-DEGs and TCGA-KIRC-down-DEGs. The lower half is interaction of SLC27A2-down-DEGs and TCGA-KIRC-up-DEGs. **F** The Kaplan–Meier curves of CDK3 in ccRCC for overall survival (OS). **G** The expression level comparsion of CDK3 between tumor samples and normal samples in TCGA-KIRC cohort. **H** The protein level of CDK3 after overexpressing or knockdown SLC27A2 in 786-O and CAKI cell lines. **I** The mRNA level of CDK3 after overexpressing or knockdown SLC27A2 in 786-O and CAKI cell lines. *p < 0.5, **p < 0.01, ***p < 0.001, and ****p < 0.0001.

Since we had studied the function of SLC27A2 in vitro, further function validation were conducted in vivo. Caki cells overexpressing SLC27A2 lentivirus were seeded subcutaneously in nude mice. Tumor sizes were measured every 3 days, and subcutaneous tumors were removed from nude mice on day 21. The results showed that overexpression of SLC27A2 reduced tumor weights and volumes in vivo (Fig. 5A–C). The above experiments provided conclusive evidence that SLC27A2 inhibited the proliferation, migration, and invasion of ccRCC cells, having important implications in the process of tumor metastasis.

Gene Set Enrichment Analysis (GSEA) was used to study the biological processes involved in SLC27A2 regulation based on the TCGA database to see how SLC27A2 affected ccRCC carcinogenesis and progression (Fig. 4E). The findings demonstrated that SLC27A2 was strongly linked to EMT signaling characteristics. Thus, we speculated that SLC27A2 affected the EMT process. Study examined at the changes in numerous EMT markers in SLC27A2 overexpression cells and siSLC27A2 cells to test this theory. These results confirmed that E-cadherin expression level was higher, and Vimentin, Snail1, N-cadherin expression level were lower in 786-O and Caki cells transfected with SLC27A2 overexpression lentivirus than 786-O and Caki cells at protein levels (Fig. 4F). The opposite results were observed in siSLC27A2 cells (Fig. 4F). In summary, SLC27A2 overexpression inhibited ccRCC EMT in vitro while the knockdown of SLC27A2 in renal cancer cells promoted ccRCC EMT in vitro.

### SLC27A2 could negatively regulate the expression of CDK3 in RCC

CAKI cells with SLC27A2 overexpression were chosen for whole transcriptome sequencing to better understand the mechanism of EMT by SLC27A2. Further analyses were used to explore potential downstream genes based our seq-data (Fig. 5D). These results confirmed that 69 mRNAs were up-regulated, and 25 mRNAs were down-regulated in overexpressing SLC27A2 CAKI cells (|fold change | > 2, *P* < 0.05). Additional bioinformatics analyses were used to analyze the sequencing results among these differentially expressed genes, and alignment with the TCGA-KIRC database yielded 7 genes with biological significance (Fig. 5E). We performed overall survival analyses and found that CDK3 was the only one in above seven candidate genes significantly associated with prognosis of renal cancer patients (Figs. 5F, S2). The mRNA expression of CDK3 in renal cancer tissues was much higher than in normal tissues, consistent with the survival analysis results, indicating that CDK3 was an unfavorable biomarkers for RCC and might participate in cancer progression (Fig. 5G). Since we had found that SLC27A2 was significantly associated with EMT signaling (Fig. 4E, F), while CDK3 was previously reported to regulate the EMT signaling pathway [14], whether SLC27A2 regulated EMT processes through CDK3 needed further study. As a result, we identified CDK3 as an important downstream molecule of SLC27A2 and conducted further experiments to figure out potential mechanism. We also verified the regulatory effect of SLC27A2 on CDK3 using qRT-PCR and western blot in two renal cancer cells (Fig. 5H, I).

### CDK3 was up-regulated in RCC and promoted its metastasis

**Fig. 6:**
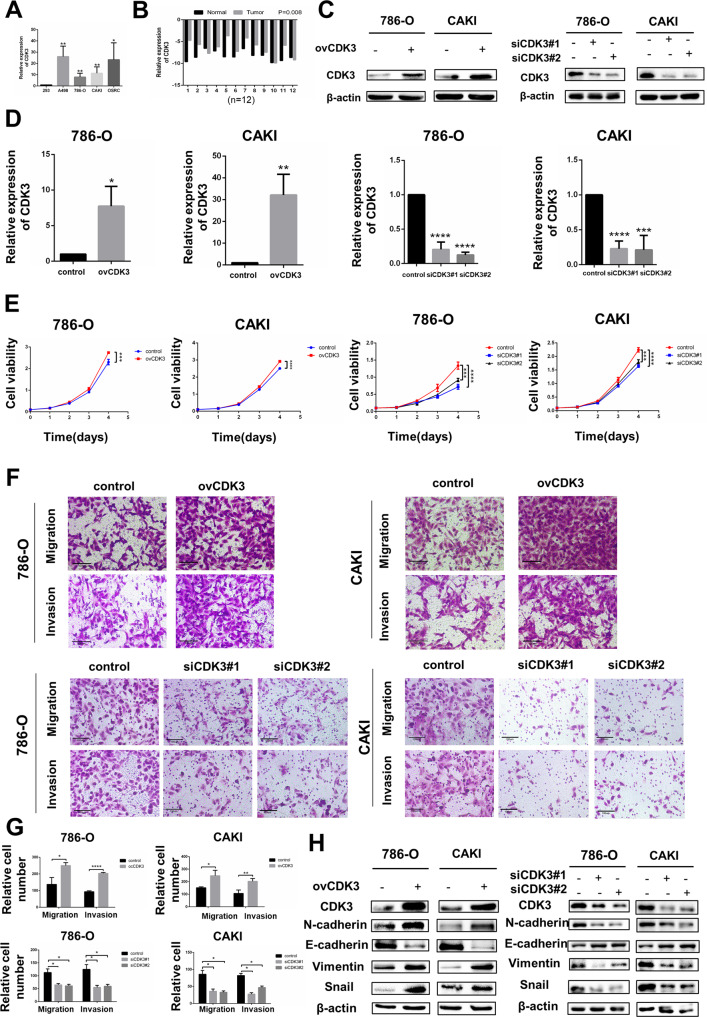
CDK3 was up-regulated in RCC and promoted its metastasis. **A** The mRNA levels of CDK3in ccRCC cells. **B** The mRNA levels of CDK3 in 12 pairs of ccRCC tissues and neighboring normal tissues. **C**, **D** CDK3 protein and mRNA expression after overexpression or knockdown. **E** Cell growth curves of CCK8 assays for indicated cells. **F**, **G** Migration and invasion assay for overexpressing or knockdown CDK3 (Magnification: 200×). *t*-test, *p < 0.05, **p < 0.01,***p < 0.001, ****p < 0.0001. **H** The expression change of EMT markers in overexpressing CDK3 cells or siCDK3 cells.

We used qRT-PCR to validate the increment of CDK3 in ccRCC cells and cancer tissues. According to these findings, the CDK3 level was considerably higher in RCC cells and cancer tissues (Fig. 6A, B) [14]. To probe the effect of CDK3 on the biological behavior of renal cell carcinoma, CDK3 plasmid was used to transfect renal cancer cells and up-regulate CDK3 expression. Compared to the negative controls, mRNA and protein expression of CDK3 in 786-O and Caki were observably higher (Fig. 6C, D). SLC27A2 small interfering RNA was used to transfect renal cancer cells and down-regulate CDK3 expression in mRNA and protein (Fig. 6C, D).

The capacity of ccRCC cells to proliferate was assessed using a CCK8 assay. These results confirmed that cells overexpressing CDK3 increased the proliferation rate of CAKI and 786-O cells significantly but knock-down of CDK3 decreased the proliferation rate of CAKI and 786-O cells (Fig. 6E). To determine the role of CDK3 in cell migration, the migratory capability of 786-ovCDK3 cells and caki-ovCDK3 cells was increased compared to the migration of control cells. The migratory capability of 786-siCDK3 cells and caki-siCDK3 cells was decreased compared to the migration of control cells. Transwell migration experiments in two ccRCC cell lines corroborated the aforesaid findings. Similarly, transwell invasion experiments were used to determine invasion capability. The potential to invade was greatly boosted in CDK3 cells that were overexpressed whereas the opposite phenomenom was observed in siCDK3 cells (Fig. 6F, G).

According to the findings, CDK3 could boost ccRCC migratory and invasive capabilities. Because EMT frequently results in the increased migratory and invasive ability of epithelial cells, and according to a previous article, CDK3 could promote the EMT process [14]. We hypothesized that CDK3 could also affect EMT processes in renal cancer. We assessed alterations in several EMT markers in CDK3-overexpressing cells to test this hypothesis. At the protein level, E-cadherin expression decreased while the expression of Vimentin, Snail1, and N-cadherin increased in 786-O and Caki transfected with CDK3 overexpression plasmid. However, in siCDK3 cells, the protein level changes of the above EMT markers were reversed (Fig. 6H). As a result, overexpression of CDK3 promoted EMT process of ccRCC in vitro while knockdown of CDK3 could inhibit EMT process.

### SLC27 A2 promoted EMT signaling and ccRCC metastasis in a CDK3-mediated manner

**Fig. 7:**
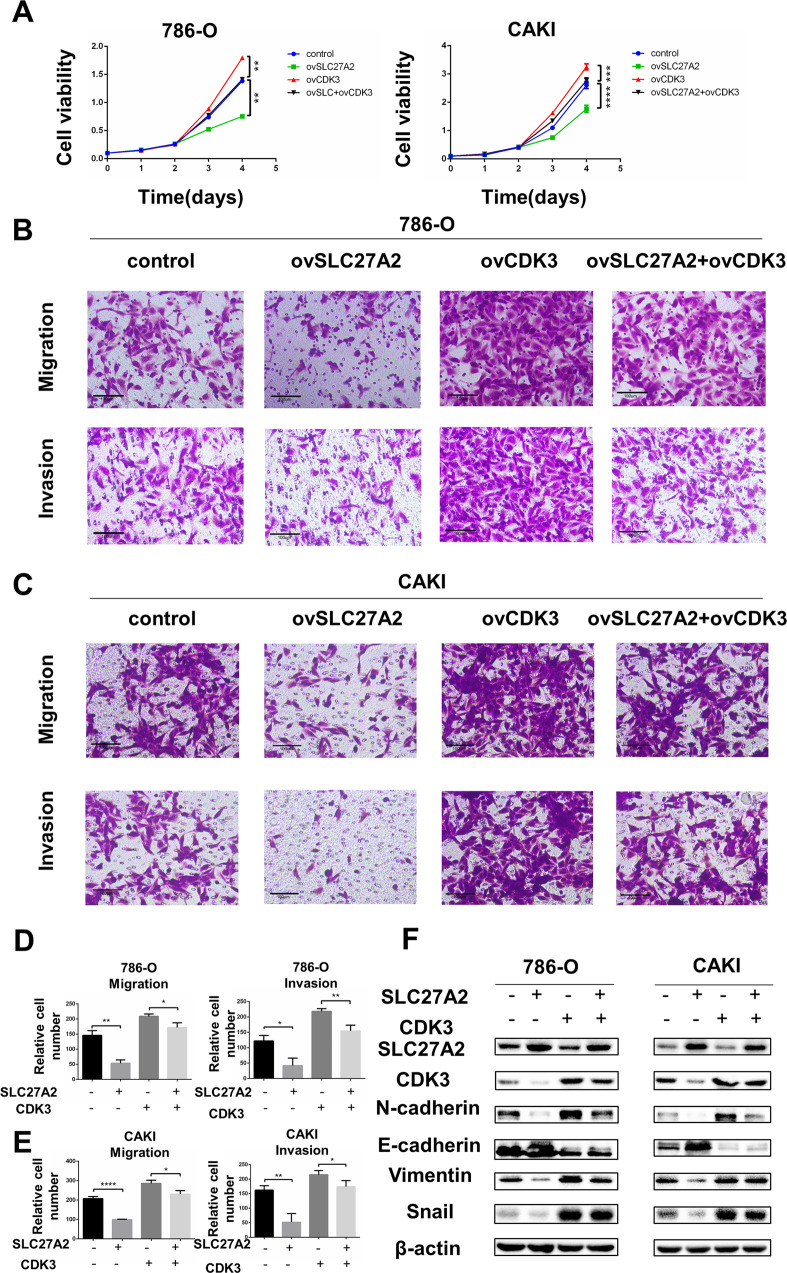
SLC27A2 promoted EMT signaling and ccRCC metastasis in a CDK3-mediated manner. **A** Cell growth curves of CCK8 assays for indicated cells. **B**–**E** Migration and invasion assay for indicated ccRCC cells (Magnification: 200×). *t*-test, *p < 0.05, **p < 0.01,***p < 0.001, ****p < 0.0001. **F** The expression change of EMT markers in indicated cells.

Based on the above experimental results, we proposed that SLC27A2 influences ccRCC development by affecting CDK3-dependent EMT processes. To demonstrate the relevance of the SLC27A2-CDK3 axis in ccRCC, a functional rescue test was carried out. Using previous methods, we constructed four sets of cell lines for rescue experiments, cell lines with control group, cell lines with overexpression SLC27A2 group, cell lines with overexpression CDK3 group, and cell lines with overexpression SLC27A2 and CDK3. As mentioned, overexpression of SLC27A2 can greatly reduce the capacity of ccRCC cells to proliferate. The CCK8 experiment revealed that overexpressing CDK3 could reduce the SLC27A2-induced suppression of cell growth to some extent (Fig. 7A). Similarly, we also constructed four cell lines for rescue experiments using small interfering RNAs, cell lines with control group, cell lines with siSLC27A2 group, cell lines with siCDK3 group, and cell lines with siSLC27A2 and siCDK3. The CCK8 experiment revealed that siCDK3 could reduce the siSLC27A2-induced cell growth to some extent (Fig. S3A).

Using transwell experiments to determine migration and invasion potential. Similarly, overexpression of CDK3 could partially reverse the SLC27A2-induced inhibition of migration and invasion (Fig. 7B–E). And siCDK3 could partially reverse the siSLC27A2-induced promotion of migration and invasion (Fig. S3B–E). As a result, a consistent conclusion about the effects of EMT signaling on ccRCC could be drawn. Overexpression of SLC27A2 alone significantly reduces epithelial-to-mesenchymal transition (EMT). As a result, overexpression of CDK3 and low expression of CDK3 can significantly promote and inhibit the transfer of the EMT pathway. To put it another way, overexpressing CDK3 could effectively counteract the physiologic consequences of SLC27A2 and knocking down CDK3 could counteract the physiologic consequences of siSLC27A2 (Fig. 7F and Fig. S3F). We could deduce that SLC27A2 inhibit ccRCC progression mainly through the CDK3-mediated EMT signaling pathway.

## Discussion

Accumulated evidence indicates that SLC27A2 is significantly underexpressed in various human malignancies, including ovarian, prostate, and lung cancers. Previous studies have shown that SLC27A2 in ovarian and lung cancers alleviates their resistance to chemotherapeutics. Conversely, SLC27A2 can promote cancer cell proliferation and migration in thyroid cancer. These findings imply that SLC27A2 may have a role in a variety of cancers. Different tumor microenvironments and genetic backgrounds may influence SLC27A2’s physiological roles in different cancers. The function of SLC27A2 in RCC, on the other hand, is unknown.

SLC27A2 was discovered to be downregulated in renal cell carcinoma tissues and cell lines, which matches numerous prior results from other cancer types. Our results suggested that SLC27A2 may inhibit migration and invasion. We used qRT-PCR and western blotting to evaluate the expression of EMT-associated markers in RCC cell lines to better know the fundamental molecular pathways by which SLC27A2 prevents RCC metastasis. The results confirmed that the protein level of epithelial cell marker E-cadherin in renal cell carcinoma cell lines was up-regulated when SLC27A2 was overexpressed. In contrast, the mesenchymal cell markers N-cadherin, Vimentin, and Snail were down-regulated when overexpressing SLC27A2, suggesting that the EMT process is inhibited during renal cell carcinoma progression. Moreover, we knocked down the expression of SLC27A2 using siRNA in renal cancer cells and observed the opposite changes of EMT markers. In vivo, overexpreesion of SLC27A2 could inhibit tumor growth. Thus, SLC27A2 may prevent RCC from migrating and invading by suppressing EMT.

The whole transcriptome sequencing showed that the increased expression of SLC27A2 can lead to the downregulation of CDK3, and it has been reported that CDK3 promotes the EMT process in colorectal cancer. Therefore, we speculated that SLC27A2 affected the EMT process through CDK3 and perform a series of experiments to validate the hypothesis. However, our study has some limitations. SLC27A2 is involved in lipid formation and fatty acid degradation and can turn free long-chain fatty acids to fatty acyl-CoA esters. Based on the prior findings of our investigation, we speculated that SLC27A2 has a regulative function in the aberrant metabolic process of ccRCC, particularly lipid metabolism. Our next study will focus on the relationship between SLC27A2 and lipid metabolism in ccRCC.

In summary, SLC27A2 expression is diminished in ccRCC, and this lower SLC27A2 expression may accelerate tumor progression through CDK3-mediated EMT. In addition, SLC27A2 has the potential to become a new diagnostic marker and prognostic factor for ccRCC, providing novel options for treating ccRCC.

## Conclusion

Our work collectively highlights the vital role of SLC27A2 in regulating EMT signaling through CDK3 in ccRCC metastasis and suggests that functional inhibition of SLC27A2-CDK3-EMT axis may be an attractive therapeutic target for metastasis of ccRCC.

## Materials and methods

### Cell culture and reagents

The American Type Culture Collection (ATCC, USA) provided the human renal carcinoma cell lines A498, 786-0, CAKI, OSRC, and 293 as well as the normal cell line 293 for this study [17]. Cells were cultured in DMEM high glucose media supplemented with 10% fetal bovine serum (FBS) and 1% penicillin-streptomycin in a 5% CO2 incubator at 37 °C [18].

### Cell infection and transfection

SLC27A2 overexpression lentivirus and the matching control vector were transfected in CAKI and 786-O cell lines by Polybrene (Genechem) and Enhanced Infection Solution (Genechem) according to the producer’s instructions. Protein lysates and total RNA were gathered 4 days after transfection for western blot and qRT-PCR verification of transfection effectiveness. When transfecting the plasmid, 3 µg of CDK3 plasmid was transfected into approximately 3 × 10^6^ cells by Lipofectamine 3000 (Invitrogen, CA, USA). 5 µg siRNA for SLC27A2 and CDK3 were transfected into approximately 3 × 10^6^ cells by Lipofectamine 3000 (Invitrogen, CA, USA). Protein lysates and total RNA were obtained 48 h after transfected for western blot and qRT-PCR verification of transfection effectiveness.

### Clinical renal cancer samples

Clinical kidney cancer samples were collected from patients in the Department of Urology, Union Hospital, Wuhan, China, who had a partial or radical nephrectomy. CcRCC samples were taken and paired with normal kidney tissues in the adjacent area. Tissue samples were frozen in −80 °C for follow-up research like RNA extractions or Western Blots. The patient had not received anticancer therapy prior to surgery. The Human Research Ethics Committee of Huazhong University of Science and Technology (Wuhan, China) approved this study and experimental protocols, and all patients provided written informed consent [18].

### IHC assay

The 4 µM formalin-fixed paraffin-embedded tissue slices were incubated for 12 h at 4 °C with a rabbit antibody against SLC27A2 (1:100) [19, 20]. The portion was then washed in PBS. 50 µl DAKO secondary antibody per section was used for immunodetection, and the sections were incubated with secondary antibodies at 25 °C for around 2 h. We chose three random fields under a light microscope at magnifications of 200× and 400×.

### RNA extraction and qRT-PCR

Total RNA was isolated from tissues and cell lines using the TRizol reagent (Thermo; Massachusetts, USA) [21, 22]. 1 μg enriched RNA was used to synthesize cDNA through reverse transcription, which the HiScript Q RT SuperMix accomplished for qPCR (Vazyme; Nanjing, CHN) [18, 23]. The HiScript II, One Step qRT-PCR SYBR Green Kit (Vazyme; Nanjing, CHN) was used for qPCR analysis by Analytikjena qPCR System (Analytikjena; GER). Samples were normalized by β-Actin.

### Western blotting experiments

Proteins from cells and tissues was obtained using radio-immunoprecipitation assay (RIPA) protein lysis buffer (Beyotime Institute of Biotechnology, Haimen, China) with added protease inhibitor cocktail and PMSF. 30 µg protein was subjected to SDS-PAGE gel [24]. Sorting the proteins was done using gel electrophoresis, and the proteins were then transferred to polyvinylidene fluoride (PVDF) membranes. The membranes were occluded at room temperature using 5% nonfat dried skimmed milk. Then, the membranes of targeted protein were reacted overnight with corresponding primary antibodies. Lastly, the membranes were removed in PBST to remove unbound antibody and then incubated with rabbit mAb or mouse mAb (HRP-conjugated Affinipure Goat Anti-Mouse IgG (H + L) and HRP-conjugated Affinipure Goat Anti-Rabbit IgG (H + L) were purchased from Proteintech and deliquated 1:3000). The antibodies used for western blots were: SLC27A2(1:2000; Abclonal, A1077), CDK3 (1:4000; Proteintech, 55103-1-AP), N-Cadherin (1:2000; Abclonal, A0433), E-Cadherin Rabbit (1:1000; Abclonal, A11509), Vimentin (1:2000; Abclonal, A11952), Snail (1:2000; Abclonal, A5243), and β-Actin (1:200000; Abclonal, AC026) [25].

### Whole transcriptome sequencing

As before reported [17, 26], total RNA was isolated by TRizol reagent (Thermo; Massachusetts, USA). The Agilent 2100 Bioanalyzer was used to assess RNA integrity (Agilent Technologies, Santa Clara, CA, USA). Following RNA extraction, purification, and library creation, paired-end sequencing on these libraries was performed using Next-Generation Sequencing technology on the Illumina sequencing platform. PANOMIX, China, supplied whole transcriptome sequencing techniques and procedures.

### Cell viability assays

2 × 10^3^ cells were grown in 96-well plates for the cell viability test. The CCK8 technique was used to determine the cell proliferation rate. Cell viability was evaluated at 0, 1, 2, 3, and 4 days upon treatments at 450 nm [27, 28].

### Migration and invasion assays

Cells were collected 2 days after transfection and resuspended in serum-free media. 3 × 10^4^ cells in serum-free medium were planted into the Boyden Transwell chambers (Corning; New York, USA) with 8-μm membrane filters for migration, whereas 6 × 10^4^ cells in serum-free medium were planted into the mentioned chambers, which were pre-coated with Matrigel (Thermo Fisher Scientific; Waltham, USA) for invasion. In advance, the chambers were placed on 24-well plates with a complete medium containing 10% FBS [29]. After 24 h at 37 °C with 5% CO2, the cells on the top portion were erased, while those on the bottom part were fixed in 100% methyl alcohol and dyed with 0.05 % crystal violet [30]. Below a microscope, cells were observed under a light microscope. All test was carried out three times in a row.

### Bioinformatics analysis

The Cancer Genome Atlas Kidney Clear Cell Carcinoma (TCGA-KIRC) database [31] was used to obtain clinical and Hiseq data used for univariate and multivariate Cox proportional hazard regression. We selected the differentially expressed genes (DEGs) of KIRC using the “limma” [32] package with parameters of adjusted *p* < 0.05 and log_2_ | FC | > 2 according to methods from previous articles [33, 34]. GSE40435 and GSE29609 data were downloaded from the NCBI-GEO database. Differential expression of SLC27A2 mRNA in three RCC datasets, including Gumz Jones and Lenberg, was obtained from the Oncomine database [35]. Detailed clinical information and characteristics of TCGA-KIRC could be downloaded from the Xena [36].

The major targets were overall survival (OS) and disease-free survival (DFS). The period from diagnosis and death, or reviewed at the last follow-up, was used to determine OS. The time between diagnosis or surgery and progression of disease or death at the last follow-up was designated as DFS. The KM curves of OS and DFS were plotted using GraphPad Prism 6.0. Overall survival analyses of seven candidate downstream genes were perfomed using online tools GEPIA [37].

According to the grouping, the GSEA software [38] was conducted for enrichment analysis. The relevant enriched pathways were thought to be *p* < 0.05, and the false discovery rate (FDR) value < 0.25.

### Statistical analysis

Excel 2016 (Microsoft) and SPSS 22.0 were used for all statistical analyses (IBM SPSS, Chicago, IL). All in vitro studies were repeated for three times, and all data were reported as mean SEM. The Student’s *t*-test and Pearson correlation coefficient were used in the statistical analysis. The significance value was determined with *p* < 0.05.

### In vivo tumor transplantation

There were 6 male nude mice in both the experimental group and the control group. 2 × 10^6^ tumor cells (Caki) were subcutaneously injected into each nude mouse. We measured tumor size every 3 days, mice were sacrificed at day 21, tumor sizes and weights were measured. All animal experiments were approved by the Institutional Animal Use and Care Committee of Tongji Medical College, Huazhong University of Science and Technology.

## Supplementary information

Supplemental Figure 1

Supplemental Figure 2

Supplemental Figure 3

supplementary legends

authors agreement

Original western blots

## Data Availability

The public datasets used in the current study can be obtained from TCGA-KIRC, Xena websites and NCBI-GEO databases. The RNA sequence data about SLC27A2 in two cell lines can be obtained from the corresponding author upon reasonable request.
