# Supplementary figures and images for: Up-regulation of SLC27A2 suppresses the proliferation and invasion of renal cancer by down-regulating CDK3-mediated EMT
Source: Cell Death Discov. 2022 Aug 4;8:351. doi: 10.1038/s41420-022-01145-8 (PMC9352701; doi:10.1038/s41420-022-01145-8)

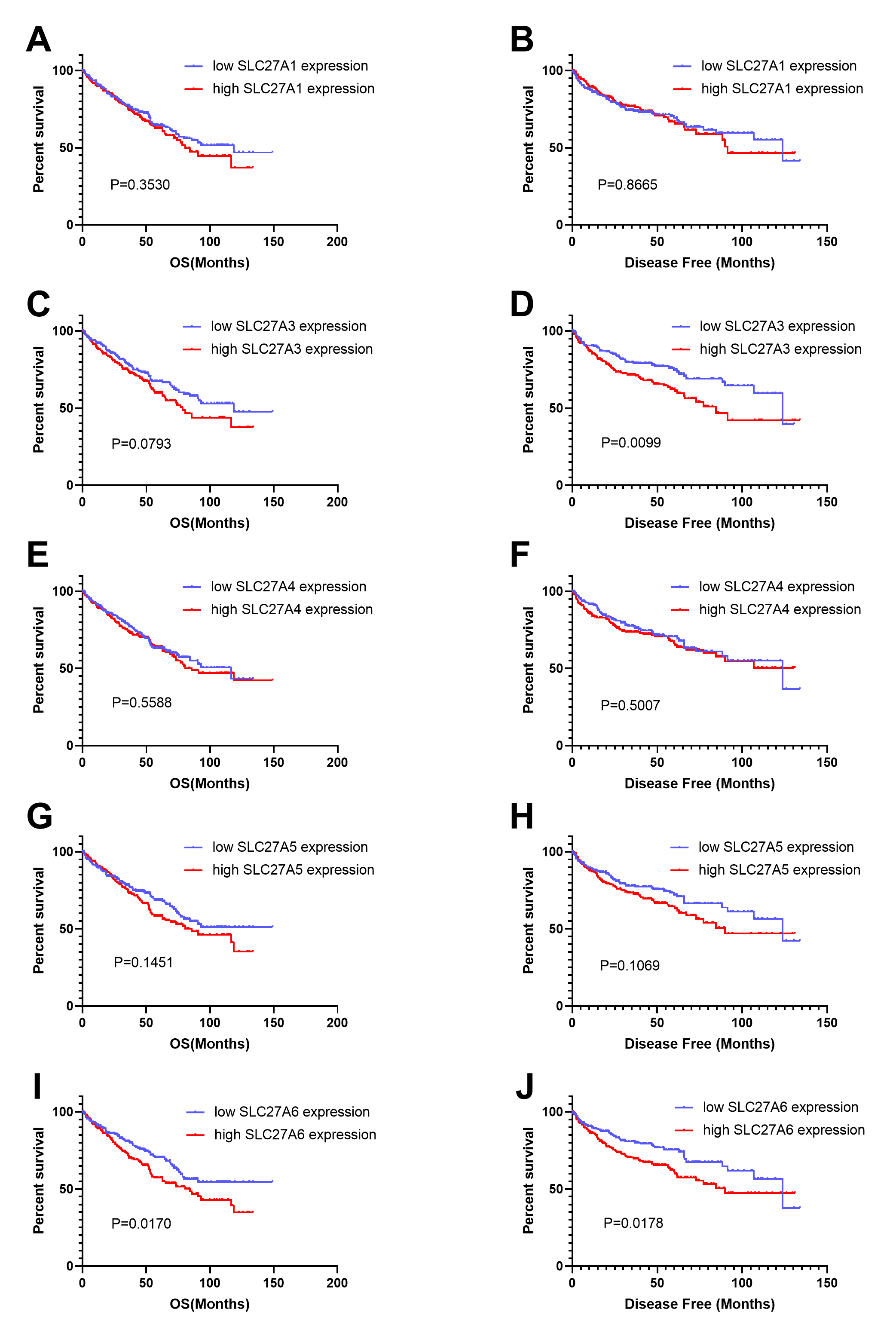

Supplement: Supplementary file 1 — Supplemental Figure 1 [file 41420_2022_1145_MOESM1_ESM.tif]

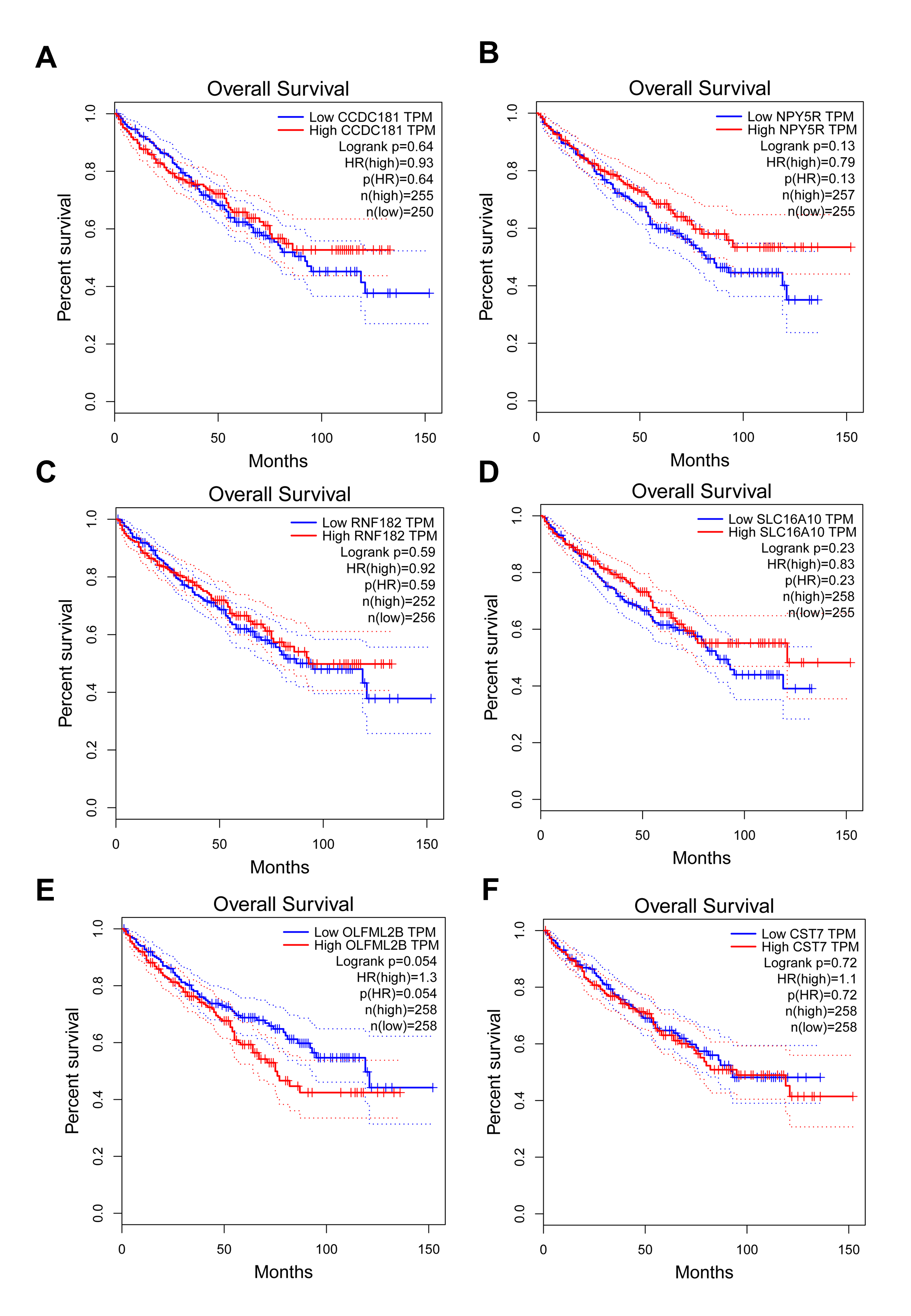

Supplement: Supplementary file 2 — Supplemental Figure 2 [file 41420_2022_1145_MOESM2_ESM.tif]

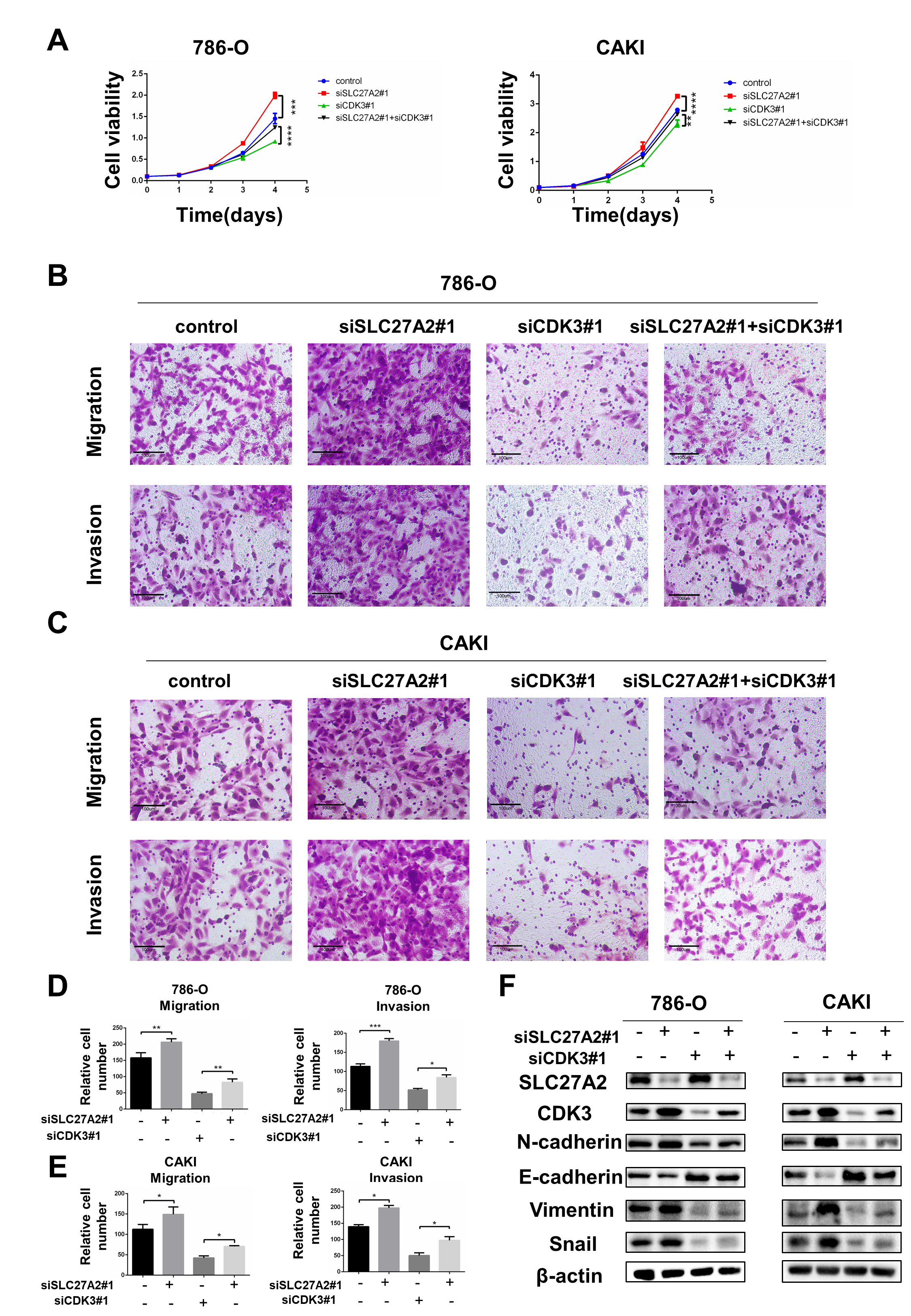

Supplement: Supplementary file 3 — Supplemental Figure 3 [file 41420_2022_1145_MOESM3_ESM.tif]

Figure 3A

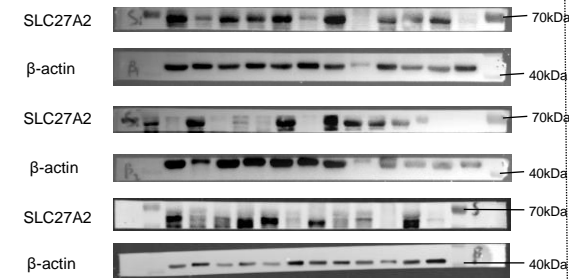

Figure 3F

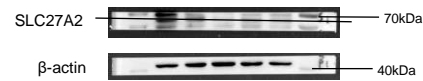

Figure 4A

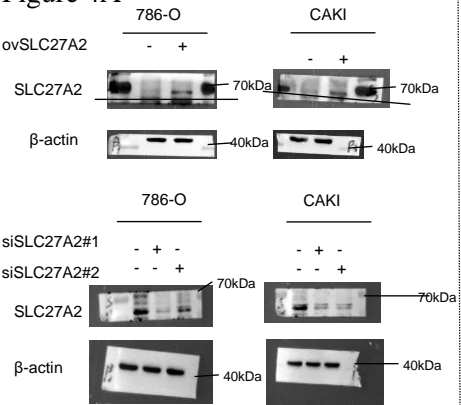

Figure 4F

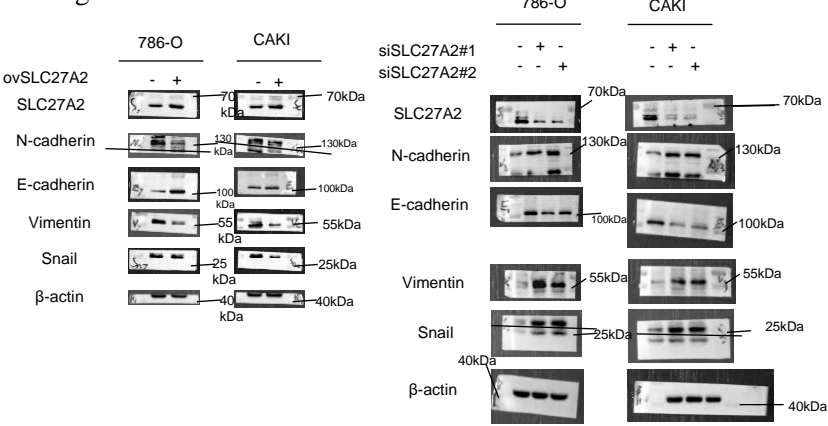

Figure 5F

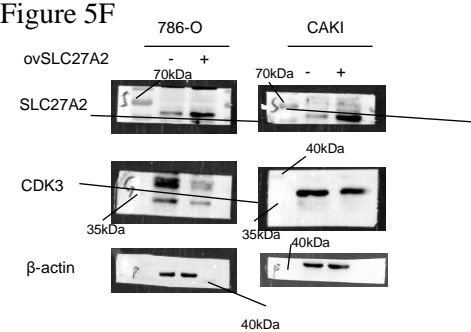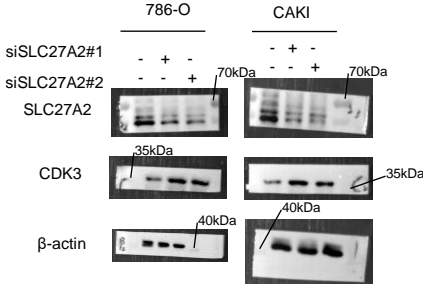

Figure 6C

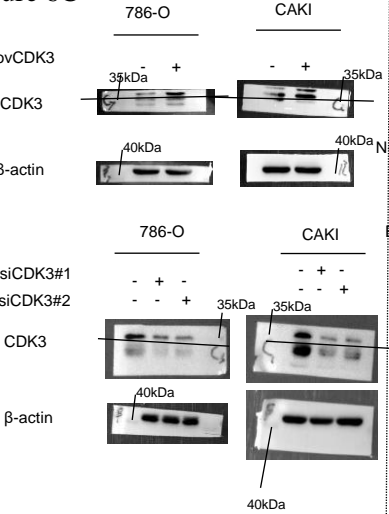

Figure 6H

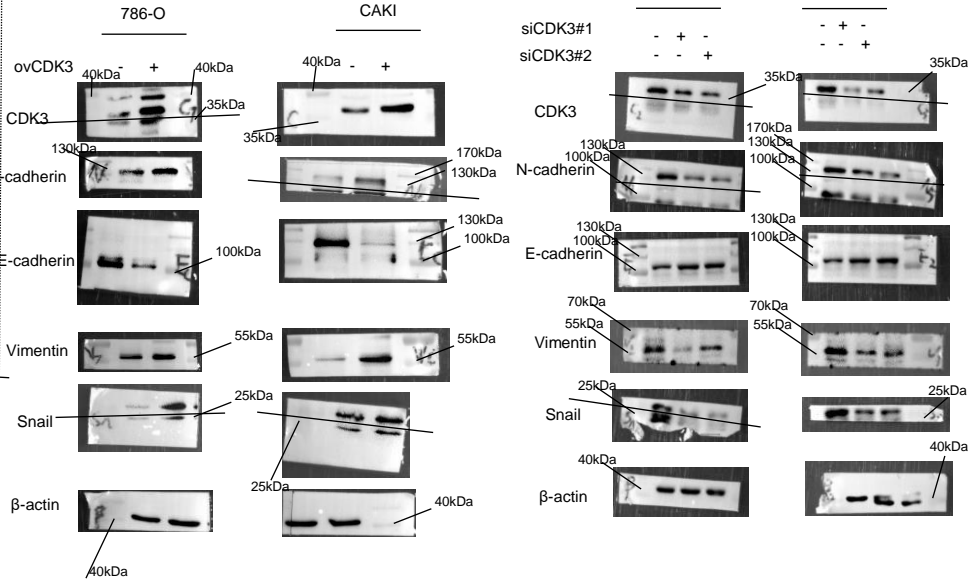

Figure 7F

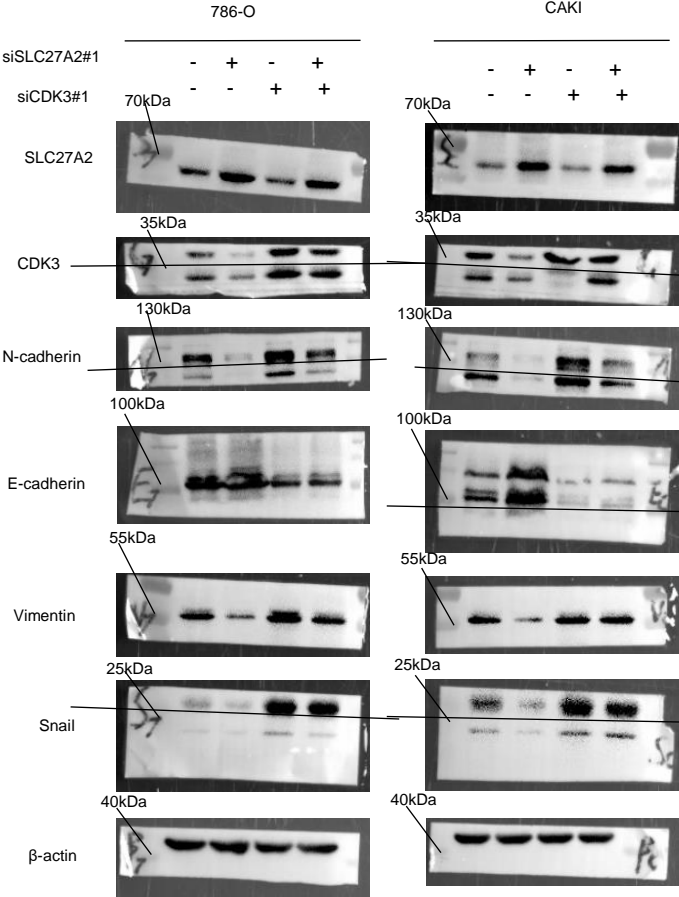

Figure S3 F

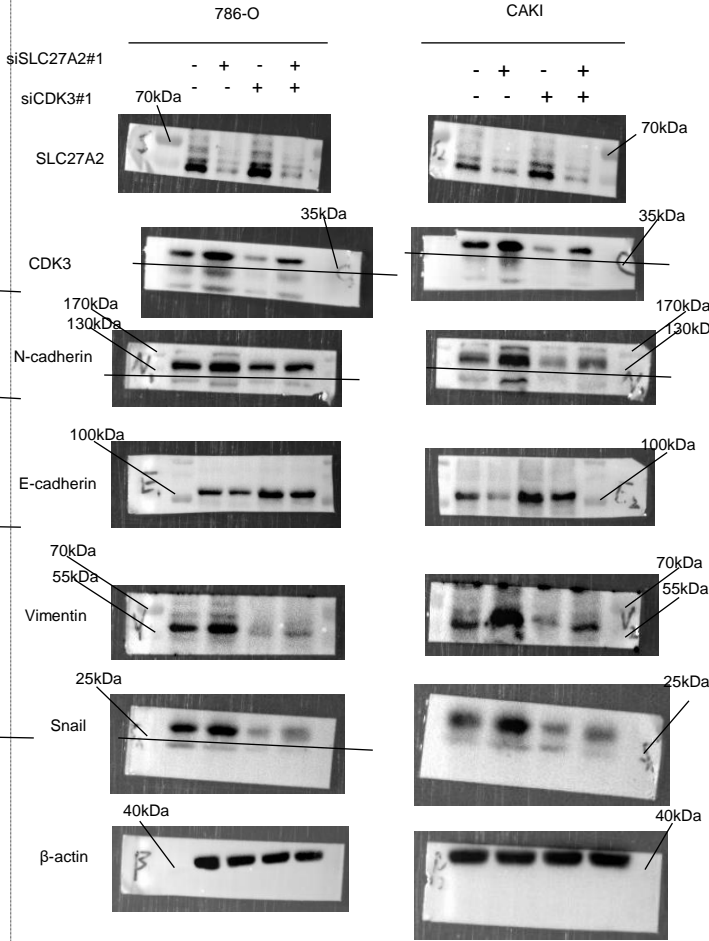

Supplement: Supplementary file 6 — Original western blots [file 41420_2022_1145_MOESM6_ESM.pdf]
