# Supplementary material for: Up-regulation of SLC27A2 suppresses the proliferation and invasion of renal cancer by down-regulating CDK3-mediated EMT
Source: Cell Death Discov. 2022 Aug 4;8:351. doi: 10.1038/s41420-022-01145-8 (PMC9352701; doi:10.1038/s41420-022-01145-8)
Supplement: Supplementary file 4 — supplementary legends [file 41420_2022_1145_MOESM4_ESM.docx]

**Supplementary legends**

**Figure S1: Kaplan-Meier curves of SLC27As expression.**

(A) and (B) SLC27A1. (C) and (D) SLC27A3. (E) and (F) SLC27A4. (G) and (H) SLC27A5. (I) and (J) SLC27A6.

**Figure S2：The Kaplan-Meier curves of interaction between SLC27A2-DEGs and TCGA-KIRC-DEGs in ccRCC for overall survival (OS).**

(A) CCDC181. (B) NPY5R. (C) RNF182. (D) SLC16A10. (E) OLFML2B. (F) CST7.

**Figure S3：SLC27A2 promoted EMT signaling and ccRCC metastasis in a CDK3-mediated manner.** (A) Cell growth curves of CCK8 assays for indicated cells. (B) and (D) Migration and invasion assay for indicated 786-O cells (Magnification: 200×). (C) and (E) Migration and invasion assay for indicated CAKI cells (Magnification: 200×). t-test, *p< 0.05, **p< 0.01,***p< 0.001, ****p< 0.0001. (F) The expression change of EMT markers in indicated cells.
