# Supplementary material for: Up-regulation of SLC27A2 suppresses the proliferation and invasion of renal cancer by down-regulating CDK3-mediated EMT
Source: Cell Death Discov. 2022 Aug 4;8:351. doi: 10.1038/s41420-022-01145-8 (PMC9352701; doi:10.1038/s41420-022-01145-8)
Supplement: Supplementary file 5 — authors agreement [file 41420_2022_1145_MOESM5_ESM.pdf]

回复：无主题

wangxuegang@xmu.edu.cn 发送给 章小平

发起会议

2022-07-19 21:28:26

I agree with the author's changes. Thank you.
Yours sincerely,
Xuegang Wang
2022.7.19

—— 回复的原邮件 ——

发件人	章小平<xzhang@hust.edu.cn>
日期	2022年07月19日 21:05
收件人	wangxuegang@xmu.edu.cn<wangxuegang@xmu.edu.cn>
主题	(无主题)

Dear Xuegang Wang,

I am very glad that our manuscript was accepted by Cell Death & Discovery. Professer Wang gave great help in the process of manuscript revision. Through all author discussions, Wang was listed as one of the corresponding authors. List of all authors as follows: Ning Xu#, Wen Xiao#, Xiangui Meng, Weiquan Li, Xuegang Wang,*, Xiaoping Zhang*, Hongmei Yang,*. Thank you very much for your efforts in the process of experimental design, manuscript writing, submission and revision. Please reply to the author changes, thank you.

Yours sincerely,

Xiaoping Zhang

2022.7.19

Re:

HONGMEI YANG 发送给 章小平

发起会议

2022-07-19 21:29:34

Dear Zhang,

I agree with the author list and order of the author changes, thank you.

Yours sincerely,

Hongmei Yang

——原始邮件——
发件人："章小平" <xzhang@hust.edu.cn>
发送时间：2022-07-19 21:13:46 (星期二)
收件人： hyang@hust.edu.cn
抄送：
主题：

Dear Hongmei Yang,

I am very glad that our manuscript was accepted by Cell Death & Discovery. Professer Wang gave great help in the process of manuscript revision. Through all author discussions, Wang was listed as one of the corresponding authors. List of all authors as follows: Ning Xu#, Wen Xiao#, Xiangui Meng, Weiquan Li, Xuegang Wang,*, Xiaoping Zhang*, Hongmei Yang,*. Thank you very much for your efforts in the process of experimental design, manuscript writing, submission and revision. Please reply to the author changes, thank you.

Yours sincerely,

Xiaoping Zhang

2022.7.19

收件箱 Re:Author ... ×

回复 回复全部 转发 移动到 标记为 更多 删除

Re:Author changes

肖文 发送给 章小平

2022-07-19 21:48:57

Dear Zhang：

According to the author' discussion, I unanimously agreed with the author change.

Yours sincerely,

Wen Xiao

2022.7.19

收件箱 Re:Author ... ×

回复 回复全部 转发 移动到 标记为 更多 删除

Re:Author changes

肖文 发送给 章小平

2022-07-19 21:48:57

xiaowenx11@163.c...

xiaowenx11@163.com

往来邮件 编辑联系人 更多

usly agreed with the author change.

Wen Xiao

2022.7.19

At 2022-07-19 21:46:10, "章小平" <xzhang@hust.edu.cn> wrote:

Dear Wen Xiao,

I am very glad that our manuscript was accepted by Cell Death & Discovery. Professer Wang gave great help in the process of manuscript revision. Through all author discussions, Wang was listed as one of the corresponding authors. List of all authors as follows: Ning Xu#, Wen Xiao#, Xiangui Meng, Weiquan Li, Xuegang Wang,, Xiaoping Zhang*, Hongmei Yang,*. Thank you very much for your efforts in the process of experimental design, manuscript writing, submission and revision. Please reply to the author changes, thank you.*

Yours sincerely,

Xiaoping Zhang

2022.7.19

回复：(无主题)

Weiquan Li 发送给 章小平

发起会议
2022-07-19 22:03:28

Dear Professor Zhang

Thank you for your letter. I totally agree with the decision since we had discussed it before.

Best wishes,
Weiquan Li
2022.7.19

—原始邮件—

发件人： "章小平"<xzhang@hust.edu.cn>
发送时间： 2022年7月19日(周二) 晚上9:17
收件人： "3465632270"<3465632270@qq.com>;
主题： (无主题)

Dear Weiquan Li,

I am very glad that our manuscript was accepted by Cell Death & Discovery. Professer Wang gave great help in the process of manuscript revision. Through all author discussions, Wang was listed as one of the corresponding authors. List of all authors as follows: Ning Xu#, Wen Xiao#, Xiangui Meng, Weiquan Li, Xuegang Wang,*, Xiaoping Zhang*, Hongmei Yang,*. Thank you very much for your efforts in the process of experimental design, manuscript writing, submission and revision. Please reply to the author changes, thank you.

Yours sincerely,

Xiaoping Zhang

2022.7.19

回复：(无主题)

Weiquan Li 发送给 章小平

发起会议
2022-07-19 22:03:28

3465632270@qq.c...
3465632270@qq.com

往来邮件 | 添加到个人通讯录 | 更多

D[illegible] totally agree with the decision since we had discussed it before.

—原始邮件—

发件人： "章小平"<xzhang@hust.edu.cn>
发送时间： 2022年7月19日(周二) 晚上9:17
收件人： "3465632270"<3465632270@qq.com>;
主题： (无主题)

Dear Weiquan Li,

I am very glad that our manuscript was accepted by Cell Death & Discovery. Professer Wang gave great help in the process of manuscript revision. Through all author discussions, Wang was listed as one of the corresponding authors. List of all authors as follows: Ning Xu#, Wen Xiao#, Xiangui Meng, Weiquan Li, Xuegang Wang,*, Xiaoping Zhang*, Hongmei Yang,*. Thank you very much for your efforts in the process of experimental design, manuscript writing, submission and revision. Please reply to the author changes, thank you.

Yours sincerely,

Xiaoping Zhang

2022.7.19

回复:

萌胖 发送给 xzhang

2022-07-19 22:06:10

Dear Zhang,

I am very grateful to Professor Wang for his contribution in the process of manuscript revision. Yes, I agree with the author changes. Thank you very much.

Yours sincerely,
Xiangui Meng
July 19, 2022

------------------ 原始邮件 ------------------

发件人: "章小平" <xzhang@hust.edu.cn>;
发送时间: 2022年7月19日(星期二) 晚上9:11
收件人: "萌胖"<2576452512@qq.com>;
主题: (无主题)

Dear Xiangui Meng,

I am very glad that our manuscript was accepted by Cell Death & Discovery. Professer Wang gave great help in the process of manuscript revision. Through all author discussions, Wang was listed as one of the corresponding authors. List of all authors as follows: Ning Xu#, Wen Xiao#, Xiangui Meng, Weiquan Li, Xuegang Wang,*, Xiaoping Zhang*, Hongmei Yang,*. Thank you very much for your efforts in the process of experimental design, manuscript writing, submission and revision. Please reply to the author changes, thank you.

Yours sincerely,
Xiaoping Zhang
2022.7.19

回复:

萌胖 发送给 xzhang

2022-07-19 22:06:10

2576452512@qq.c...
2576452512@qq.com

往来邮件 | 添加到个人通讯录 | 更多

ontribution in the process of manuscript revision. Yes, I agree with the author changes. Thank you very much.

------------------ 原始邮件 ------------------

发件人: "章小平" <xzhang@hust.edu.cn>;
发送时间: 2022年7月19日(星期二) 晚上9:11
收件人: "萌胖"<2576452512@qq.com>;
主题: (无主题)

Dear Xiangui Meng,

I am very glad that our manuscript was accepted by Cell Death & Discovery. Professer Wang gave great help in the process of manuscript revision. Through all author discussions, Wang was listed as one of the corresponding authors. List of all authors as follows: Ning Xu#, Wen Xiao#, Xiangui Meng, Weiquan Li, Xuegang Wang,*, Xiaoping Zhang*, Hongmei Yang,*. Thank you very much for your efforts in the process of experimental design, manuscript writing, submission and revision. Please reply to the author changes, thank you.

Yours sincerely,
Xiaoping Zhang
2022.7.19

回复:

徐凝 发送给 章小平

2022-07-19 22:11:42

Dear Zhang,

I agree with the above author changes, thanks.

Yours sincerely,
Ning Xu
2022. 07. 19

------------------ 原始邮件 ------------------

发件人: "章小平" <xzhang@hust.edu.cn>;
发送时间: 2022年7月19日(星期二) 晚上9:12
收件人: "徐凝"<972784964@qq.com>;
主题: (无主题)

Dear Ning Xu,

I am very glad that our manuscript was accepted by Cell Death & Discovery. Professer Wang gave great help in the process of manuscript revision. Through all author discussions, Wang was listed as one of the corresponding authors. List of all authors as follows: Ning Xu#, Wen Xiao#, Xiangui Meng, Weiquan Li, Xuegang Wang,*, Xiaoping Zhang*, Hongmei Yang,*. Thank you very much for your efforts in the process of experimental design, manuscript writing, submission and revision. Please reply to the author changes, thank you.

Yours sincerely,

Xiaoping Zhang

2022. 7. 19

回复:

徐凝 发送给 章小平

2022-07-19 22:11:42

972784964@qq.com
972784964@qq.com

往来邮件 | 添加到个人通讯录 | 更多

ges, thanks.

------------------ 原始邮件 ------------------

发件人: "章小平" <xzhang@hust.edu.cn>;
发送时间: 2022年7月19日(星期二) 晚上9:12
收件人: "徐凝"<972784964@qq.com>;
主题: (无主题)

Dear Ning Xu,

I am very glad that our manuscript was accepted by Cell Death & Discovery. Professer Wang gave great help in the process of manuscript revision. Through all author discussions, Wang was listed as one of the corresponding authors. List of all authors as follows: Ning Xu#, Wen Xiao#, Xiangui Meng, Weiquan Li, Xuegang Wang,*, Xiaoping Zhang*, Hongmei Yang,*. Thank you very much for your efforts in the process of experimental design, manuscript writing, submission and revision. Please reply to the author changes, thank you.

Yours sincerely,

Xiaoping Zhang

2022. 7. 19
